# Comparison of 3D anatomical dose verification and 2D phantom dose verification of IMRT/VMAT treatments for nasopharyngeal carcinoma

**DOI:** 10.1186/1748-717X-9-71

**Published:** 2014-03-07

**Authors:** Hailei Lin, Shaomin Huang, Xiaowu Deng, Jinhan Zhu, Lixin Chen

**Affiliations:** 1 Department of Radiation Oncology, Sun Yat-Sen University Cancer Center, State Key Laboratory of Oncology in Southern China, Collaborative Innovation Center for Cancer Medicine, Guangzhou 510060, China; 2 Department of Radiation Oncology, Beijing Hospital of the Ministry of Health, Beijing 100730, China; 3 School of Physics and Engineering, Sun Yat-sen University, Guangzhou 510275, China

**Keywords:** IMRT, VMAT, 3D anatomical dose, 2D phantom dose, Dosimetry verification

## Abstract

**Background:**

The two-dimensional phantom dose verification (2D-PDV) using hybrid plan and planar dose measurement has been widely used for IMRT treatment QA. Due to the lack of information about the correlations between the verification results and the anatomical structure of patients, it is inadequate in clinical evaluation. A three-dimensional anatomical dose verification (3D-ADV) method was used in this study to evaluate the IMRT/VMAT treatment delivery for nasopharyngeal carcinoma and comparison with 2D-PDV was analyzed.

**Methods:**

Twenty nasopharyngeal carcinoma (NPC) patients treated with IMRT/VMAT were recruited in the study. A 2D ion-chamber array was used for the 2D-PDV in both single-gantry-angle composite (SGAC) and multi-gantry-angle composite (MGAC) verifications. Differences in the gamma pass rate between the 2 verification methods were assessed. Based on measurement of irradiation dose fluence, the 3D dose distribution was reconstructed for 3D-ADV in the above cases. The reconstructed dose homogeneity index (HI), conformity index (CI) of the planning target volume (PTV) were calculated. Gamma pass rate and deviations in the dose-volume histogram (DVH) of each PTV and organ at risk (OAR) were analyzed.

**Results:**

In 2D-PDV, the gamma pass rate (3%, 3 mm) of SGAC (99.55% ± 0.83%) was significantly higher than that of MGAC (92.41% ± 7.19%). In 3D-ADV, the gamma pass rates (3%, 3 mm) were 99.75% ± 0.21% in global, 83.82% ± 16.98% to 93.71% ± 6.22% in the PTVs and 45.12% ± 32.78% to 98.08% ± 2.29% in the OARs. The maximum HI increment in PTVnx was 19.34%, while the maximum CI decrement in PTV1 and PTV2 were -32.45% and -6.93%, respectively. Deviations in dose volume of PTVs were all within ±5%. D2% of the brainstem, spinal cord, left/right optic nerves, and the mean doses to the left/right parotid glands maximally increased by 3.5%, 6.03%, 31.13%/26.90% and 4.78%/4.54%, respectively.

**Conclusion:**

The 2D-PDV and global gamma pass rate might be insufficient to provide an accurate assessment for the complex NPC IMRT operation. In contrast, the 3D-ADV is superior in clinic-related quality assurance offering evaluation of organ specific pass rate and dose-volume deviations.

## Background

Intensity-modulated radiation therapy (IMRT) and volumetric modulated arc therapy (VMAT) techniques are able to provide very high dose conformity for cancer radiotherapy; thus, the surrounding normal tissue and organs can be well protected when high-dose radiation is delivered to the target volume. However, many uncertainties exist in the treatment planning and operation process that can lead to deviations of the IMRT or VMAT dose distribution. Therefore, it is necessary to verify the irradiation dose distribution that is delivered by the accelerator before such kinds of treatments [1]. Until recently, a hybrid plan has been adopted most often in 2-dimensional (2D) planar dose measurement verifications. The precision of irradiating doses is evaluated and verified by comparing the planned dose distribution, calculated by the treatment planning system (TPS), to the measured results [2]. However, due to the lack of information about the correlations between the verification measurement results and the anatomical structure of patients, as well as the resulting lack of information about the actual irradiation doses to different target volumes and organs at risk (OARs), it is difficult to identify the geometric locations where dose errors occur during plan implementation, thus leading to inadequate clinical evaluation information [3]. Recently, some 3-dimensional (3D) dose verification tools that provide patient anatomical structure information were applied clinically. These tools can provide important information such as the dose deviations, the pass rates and the locations of the dose deviations in the patients’ target volumes and organs, as well as identification of the error origins [4,5]. In this study, we adopted a 3D anatomical dose verification (3D-ADV) based on measurements of delivered dose fluence and patients’ anatomical images. Meanwhile, the traditional 2D phantom verification (2D-PDV) using an ionization chamber array with angular response correction applied an in-house software, were used to compare the efficacies of dose verification for IMRT and VMAT of nasopharyngeal carcinoma (NPC). As a result, the differences between the two verification methods and their clinical significances in evaluations of irradiating dose deviations were analyzed and clarified.

## Materials and methods

### Dose verification tools

A commercialized 3D dose verification system [6] (COMPASS, IBA Dosimetry, Schwarzenbruck, Germany) was used. The system included an online 2-dimensional ion-chamber array (2D-IC array) and dose reconstruction software based on a beam model describing the characteristics of the accelerator (e.g., energy spectrum, lateral beam quality variations) and the collapsed-cone convolution/superposition (CC) algorithm, which computed the radiation dose distribution on to the patient CT image set. A strict commissioning of the whole system, including the validation of accuracy for 2D-IC array measurement, beam modeling and dose reconstruction, was performed in advance according to the same standards as the clinic used TPS. In this previously commissioning job, which had been published [7], phantom plans of regular, irregular fields and IMRT were selected tested. All test plans were implemented and the dose distributions were measured using thimble ion-chamber and the 2D-IC array, the accuracy of the 3D-ADV system were then evaluated by comparing the corresponding measurement results. The average deviation of the 3D-ADV system was less then 1% with the largest difference of 2.12% comparing to the thimble ion-chamber measurements. In the comparison of IMRT planning calculation and the 3D-ADV system computation, the global and PTV gamma pass rates were better than 99% and 98%, respectively.

**Figure 1:**
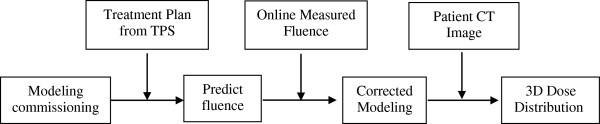
Workflow chart of the 3D-ADV system.

In the clinic IMRT dose verification practice, the actual radiation fluence delivered by accelerator was measured with the online 2D-IC array, and applied to correct the original beam model for establishing the reconstructed dose distribution (RDD) on the patient’s anatomical image, which was then compared with the therapy plan to obtain the verification results (Figure 1).

The 2D-IC array (MatriXX, IBA Dosimetry) used in this study comprised 1020 plane-parallel ion-chambers arranged with a distance of 0.762 cm between the chamber centers. A build-up of 3.3-mm equivalent water thickness was placed on top of the chambers, and a 22-mm-thick RW3 backscattering phantom (composition: 98% Polystyrol, 2% TiO_2_ and density: 1.045 g/cm^3^) was placed on the back. The array was inserted into an additional phantom with exterior dimensions of 31.4 cm × 34 cm × 22 cm (MULTICube Lite, IBA Dosimetry) for multi-gantry-angle composite 2D dose verification, equipped with an angular position sensor for angular response corrections.

### Planning and delivery system

The IMRT and VMAT plans measured in this study were designed by a 3D inverse treatment planning system (Monaco 3.0, Elekta, Stockholm, Sweden). The planned dose distribution was calculated using a fast Monte Carlo algorithm (XVMC) with the variance rate of 3% and grid size of 3 mm, as same as the reported study of Boggula et al. [4]. A 6MV X-ray irradiation was delivered via a linear accelerator (Synergy VMAT, Elekta) that supported step-and-shoot IMRT and VMAT therapies.

### Methods of dose verification

#### 2D-PDV

The above-mentioned MatriXX 2D-IC array with the MULTICube phantom was used to perform the 2D-PDV for 20 NPC treatment cases, 10 cases treated with IMRT and 10 cases with VMAT. The tests were performed in single-gantry-angle composite (0°, SGAC) and multi-gantry-angle composite (MGAC) methods, respectively [8]. Different from the published method of angle dependence correction for MatriXX [9], an in-house correction software program was used to compute the angular correction for each independent ion-chamber of the 2D-IC array in the MGAC measurements. A comparison was performed to identify the differences in the gamma pass rates (3%, 3 mm) [10] at the isocenter plane from the 2 composite dose verifications.

#### Angle correction of the ion-chamber detector

Within a gantry angle range from 0° to 360° with a step size of 5° (a step size of 1° within the range of 90° ± 5° and 270° ± 5°), the dose at the point of central detector was calculated by TPS and measured when the gantry was maintained at the same position. The correction factor of the central detector at each angle was then obtained by comparing the difference between the measurement and TPS calculation, and was normalized to the one at zero degree gantry angle. These correction factors can be applied to all the other ion-chambers in the array assuming the angular response coincidence is high among all of them. Due to the differences in the incident angles of the central and the other ion-chambers, the incident angle of each chamber was calculated with the following formula. For an angle other then the above tested ones, a linear interpolation method was adopted to get the corresponding angular correction factor. Visual C++ language was used to program this angle correction for the measurement results.

The formula for calculating the incident angle of the ion-chamber is as following.

$$\phi =\arctan\frac{d+\mathrm{SAD}\cdot \sin\theta }{\mathrm{SAD}\cdot \cos\theta }$$

**Figure 2:**
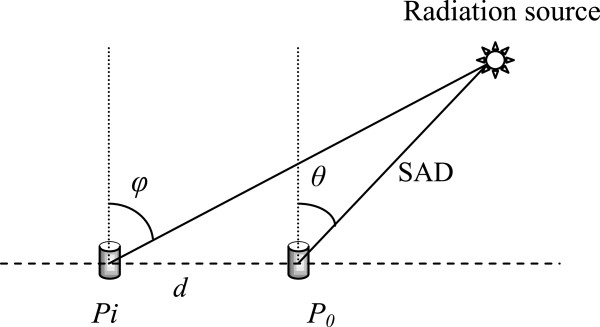
**The incident angle of every detector in the ion-chamber array used for angular correction, *Pi *** **is the position of detector *i *** **in a raw while *P***_***0 ***_**is the center of the raw.**

As shown in Figure 2, *φ* is the incident angle of each ion-chamber, *θ* is the incident angle of the center of the ion-chamber array, *SAD* is the source axis distance and *d* is the distance from each ion-chamber to the center of the ion-chamber array in each row.

#### 3D-ADV

3D-ADV test was done for the same 20 NPC IMRT/VMAT plans as well, with the 3D dose verification system that passed the commissioning test in advance as briefly described above, similar with that reported by Boggula et al. [11]. During the plan delivery, the irradiated fluence of every radiation beam was measured by the 2D-IC array mounted on a holder attached to the radiation head, perpendicular to the radiation beam. According to the measured results, the dose distribution calculation was corrected and the final dose reconstruction (RDD) was obtained for each plan case; subsequently, the RDD results were compared to the original treatment plan. Parameters such as the homogeneity index (HI) and the conformity index (CI) of the planning target volumes for primary nasopharynx tumor (PTVnx), high-risk subclinical region (PTV1), and the preventive irradiation region (PTV2), the dose volume parameters of every PTV and OAR and the gamma pass rate (3%, 3 mm) for global and for each organ were analyzed.

The formula for calculating the HI and CI of the PTV:

$$\mathrm{CI}=\frac{\mathrm{PTV}95\%}{\mathrm{PTV}}\times \frac{\mathrm{PTV}95\%}{V95\%}$$

PTV95% is the target volume that received 95% of the prescribed dose, PTV is the planning target volume and V95% is the volume that received 95% of the prescribed dose [12].

$$\mathrm{HI}=\frac{D2\%-D98\%}{D50\%}$$

D2%, D50% and D98% indicate the doses that covered 2% (near-maximum dose), 50%, and 98% (near-minimum dose) of the PTV, respectively [13].

#### Statistics

SPSS 18.0 software (Chicago, IL, USA) was used for 1 sample T-tests of the dose verification data from the 20 cases of IMRT/VMAT test plans. A value of P < 0.05 was considered statistically significant.

## Results

### Results from the SGAC/MGAC 2D-PDV

The 2D-PDV gamma pass rate of the 0° SGAC was significantly higher than that of MGAC. The average gamma pass rate (3%, 3 mm) was 99.55% ± 0.83% (96.82%-100%) in SGAC measurement; 92.41% ± 7.19% (72.3%-99.40%) and 89.22% ± 10.61% (59.74%-99.00%) in MGAC verification with the in-house and Matrixx build-in angular response correction, respectively (Table 1).

**Table 1:** The gamma pass rates (3%, 3 mm) of the 0° SGAC and MGAC in 2D-PDV

**Case #**	**0° SGAC**	**MGAC**	
		**Matrixx build-in ang corr**	**In-house ang corr**
IMRT_1_	100.00%	75.21%	85.48%
IMRT_2_	100.00%	77.89%	85.44%
IMRT_3_	100.00%	84.10%	89.47%
IMRT_4_	99.85%	84.98%	89.47%
IMRT_5_	99.07%	59.74%	72.30%
IMRT_6_	97.92%	97.06%	96.92%
IMRT_7_	99.79%	97.26%	98.35%
IMRT_8_	99.38%	87.81%	90.96%
IMRT_9_	96.82%	96.26%	94.21%
IMRT_10_	99.52%	88.71%	93.08%
VMAT_1_	100.00%	99.00%	98.33%
VMAT_2_	100.00%	98.66%	99.40%
VMAT_3_	99.06%	88.45%	91.80%
VMAT_4_	100.00%	96.02%	97.39%
VMAT_5_	100.00%	92.32%	92.72%
VMAT_6_	100.00%	96.79%	98.84%
VMAT_7_	99.53%	98.45%	98.79%
VMAT_8_	100.00%	73.66%	80.00%
VMAT_9_	100.00%	97.61%	98.67%
VMAT_10_	100.00%	94.32%	96.58%
Mean pass rate ± σ	99.55% ± 0.83%	89.22% ± 10.61%	92.41% ± 7.19%

### Comparison of 3D-ADV and TPS planning

The 3D-ADV system is able to calculate the global gamma pass rates and the pass rates in each organ of interest, respectively. In this study, the global gamma pass rate counted the area covered all the voxel in the plan CT image that the planned doses were higher than 20 cGy; while the gamma pass rate of a particular organ counted all the voxel included in the organ contour. The criterion of 3% absolute dose and 3 mm distance to agreement (DTA) was adopted in this study. The mean global gamma pass rate was 99.75% ± 0.21%. The mean gamma pass rates of the PTVnx, PTV1 and PTV2 were 83.82% ± 16.98%, 90.68% ± 9.34% and 93.71% ± 6.22%, respectively. For the OARs of brainstem, spinal cord, left and right parotid gland, the mean gamma pass rates were 96.93% ± 4.58%, 65.69% ± 20.54%, 97.33% ± 3.72% and 98.08% ± 2.29%, respectively. All the organ specific pass rates analyzed were lower than the global one (Table 2).

**Table 2:** The mean gamma pass rates (3%, 3 mm) of global volume and selected organs in 3D-ADV for the 20 NPC IMRT/VMAT plans (Std indicated the standard deviation)

**Cases**	**Global**	**PTVnx**	**PTV1**	**PTV2**	**Brainstem**	**Spinal cord**	**Parotid gland-L**	**Parotid gland-R**
Mean pass rate ± Std.	99.75 ± 0.21	83.82 ± 16.98	90.68 ± 9.34	93.71 ± 6.22	96.93 ± 4.58	65.69 ± 20.54	97.33 ± 3.72	98.08 ± 2.29

Comparing the measured RDD and the corresponding treatment plan, there were deviations at various levels in both the HI of PTVnx and the CI of the PTV1 and PTV2. The maximum deviation of the PTVnx HI from the planned value was 19.34%, and the mean deviation was -0.94% ± 9.24% (P > 0.05). The mean deviations on CI were -3.21% ± 10.53% (P > 0.05) in PTV1 with a maximum decrement of -32.45%, and -1.92% ± 3.00% (P < 0.05) with a maximum decrement of -6.93% in PTV2. Although only the CI difference in PTV2 was statistically significant, the deviations of HI in PTVnx and CI in PTV1 indicated an obvious individual difference (Table 3).

**Table 3:** The deviations in target volume HI and CI between the RDD and the plan value

**Parameters**	**PTVnx (HI)**	**PTV1 (CI)**	**PTV2 (CI)**
Δ (%)	[-15.62,19.34]	[-32.45, 13.97]	[-6.93, 2.73]
$\overset{-}{\delta }$ (%)	-0.94 ± 9.24	-3.21 ± 10.53	-1.92 ± 3.00
*P*	0.653	0.189	0.012

Note: The relative deviation between the RDD and the Plan is expressed as $\Delta =\frac{\mathrm{RDD}-\mathrm{Plan}}{\mathrm{Plan}}\times 100\%$ (RDD and Plan are the computed values from the reconstructed dose distribution and the therapy plan, respectively; Δ represents the interval range of the relative deviation). $\overset{-}{\delta }$ represents the mean value and standard deviation of the relative deviation between the RDD and Plan.

All the relative deviations in D_v_ (The absorbed dose that covers a specified fractional volume V), and V_d_ (the volume that received at least the absorbed dose d) of each target volume, between the RDD and the planned value, were within ±5%. The mean deviations in the near-minimum dose (D98%), the near-maximum dose (D2%) and the dose covering 95% of the PTV (D95%) were all less than 2%. The mean deviation in the volumes covered by 100% and 95% of prescription dose were all less than 0.5%. The results also shown that the maximum positive D2% deviations in the brainstem and spinal cord were 3.50% and 6.03% higher, respectively; the maximum positive deviations of D2% in the optic chiasm, left and right optic nerves were 26.99%, 31.13% and 26.90% higher, respectively. The maximum deviations of the mean doses (D_mean_) to the left and right parotid glands were both less than 5%. Except for the near-maximum dose (D2%) in spinal cord, there were no statistically significant discrepancies in the reconstructed dose in OARs (Table 4).

**Table 4:** The relative deviations in dose-volume histogram (DVH) parameters between those obtained from the RDD and those expected by the therapy plan

	**Parameters**	**D98%**	**D2%**	**D95%**	**D**_**mean**_	**V100%**	**V95%**
PTVnx	Δ (%)	[-1.95,4.70]	[-1.39,3.60]	[-1.55,4.72]		[-2.95,4.33]	
	$\overset{-}{\delta }$ (%)	0.93 ± 1.88	0.93 ± 1.52	1.07 ± 1.85		0.44 ± 1.72	
	*P*	0.041	0.013	0.018		0.264	
PTV1	Δ (%)	[-3.61,4.73]	[-1.34,3.54]	[-2.95,4.58]			[-0.80,1.25]
	$\overset{-}{\delta }$ (%)	0.97 ± 2.17	1.07 ± 1.44	1.01 ± 2.06			0.04 ± 0.35
	*P*	0.060	0.004	0.042			0.654
PTV2	Δ (%)	[-1.58,2.97]	[-1.93,3.71]	[-1.10,3.09]			[-0.35,0.39]
	$\overset{-}{\delta }$ (%)	0.21 ± 1.43	1.04 ± 1.57	0.54 ± 1.48			-0.05 ± 0.19
	*P*	0.526	0.010	0.132			0.268
Brainstem	Δ (%)		[-5.71,3.50]				
	$\overset{-}{\delta }$ (%)		-0.90 ± 2.22				
	*P*		0.086				
Spinal Cord	Δ (%)		[-3.36,6.03]				
	$\overset{-}{\delta }$ (%)		1.56 ± 2.03				
	*P*		0.012				
Optic Chiasm	Δ (%)		[-20.94,26.99]				
	$\overset{-}{\delta }$ (%)		-0.21 ± 11.80				
	*P*		0.939				
Optic nerve (Left)	Δ (%)		[-11.54,31.13]				
	$\overset{-}{\delta }$ (%)		0.34 ± 9.49				
	*P*		0.873				
Optic nerve (Right)	Δ (%)		[-11.36,26.90]				
	$\overset{-}{\delta }$ (%)		-0.38 ± 9.08				
	*P*		0.855				
Parotid gland (Left)	Δ (%)				[-4.49,4.78]		
	$\overset{-}{\delta }$ (%)				-0.55 ± 2.71		
	*P*				0.380		
Parotid gland (Right)	Δ (%)				[-3.15,4.54]		
	$\overset{-}{\delta }$ (%)				0.40 ± 2.00		
	*P*				0.367		

Note: Δ and $\overset{-}{\delta }$ refer to Table 3.

## Discussion

An effective evaluation of the treatment operation in clinical practice should be able to reflect the true delivery condition of the treatment and any errors occurred in the planned parameters. The traditional 2D-PDV QA, especially the SGAC measurement which used only a single fixed incident angle to avoid the existence of angular response errors, can result in inconsistencies between the QA and clinical therapy conditions [14], and not able to provide information regarding the relationship between the dose error and the anatomical structures. This leads to decreasing of QA abilities in clinical evaluations [3]. In our study of 2D-PDV of 20 NPC IMRT/VMAT plans, the gamma pass rates obtained from the SGAC verification were significantly higher than the results from the MGAC measurements done with the same gantry angle of therapy. This might be explained by the fact that the SGAC verification cannot reflect the effects on the radiation dose caused by gravity-induced changes in the multi-leaf collimator (MLC) position, or the output dose angle dependence from the accelerator under different gantry angles. The results indicated that the pass rates corrected by MGAC verification with the MatriXX build-in angle correction factor were lower than those corrected by our in-house correction software. The reason could be that the correction factor of the former was determined according to the correction angle of the central detector and then used to correct all of the other chambers without considering the differences in the incident angles of each detector. The angle correction from the in-house software considered the influence of different incident angles of each detector in the 2D-IC array; therefore, the pass rates in the verification were significantly superior to the results without the independent incident angle correction.

3D-ADV can provide us with information such as the pass rates (the global, each target and OAR volumes in the measured area), the statistical results of deviations in the dose-volume histogram (DVH) parameters (including the dose volume and the volume dose) of each organ and the anatomical positions that correspond to the dose deviations. The 3D-ADV results from the 20 nasopharyngeal carcinoma patients, who received IMRT/VMAT irradiation, revealed that the mean global gamma pass rate was 99.75% ± 0.21%. Lee et al. [15] reported that the mean gamma pass rates (3%, 3 mm) of 2D-PDV were 98.2% ± 1.3% and 98.5% ± 1.3% in nasopharyngeal carcinoma patients treated with IMRT and VMAT, respectively. Our results from the 2D-PDV with SGAC and MGAC on a similar group of patients were 99.55% ± 0.83% and 92.41% ± 7.19%, respectively. The global gamma pass rate from the 3D dose verification was similar with that from the 2D-PDV of the SGAC method but was higher than that from the 2D-PDV of MGAC measurement. The reason for the higher global pass rate in the 3D dose verification could be that, compared to 2D dose verification, the dose pixels evaluated in the 3D dose verification included all points within the CT scanning area, thus resulting in a relatively lower ratio of pixel numbers at which dose deviations occurred to the overall pixel number. Additionally, during the implementation of MGAC 2D-PDV, the pass rates might decrease due to the non-uniform phantom density that can result from the detector arrangement when the incident angles are parallel to the detector plane [16]. The gamma pass rates of each PTV and OAR decreased at different levels in all cases when compared to the global pass rate, indicating that higher gamma pass rates might be caused by improper evaluation strategies in which some errors in the delivered dose distribution were disguised and ignored due to the use of the global pass rate in the evaluation.

In this study of 3D-ADV, no statistically significant difference was found in the HI of the PTVnx between the measurement based dose reconstruction and the planned value of the TPS. However, there were obvious alterations in the verification results for each patient that the HI deviation ranged from -15.62% to 19.34%, indicating that there were significant individual differences in the irradiation results. Some patients had greater HI values in the PTVnx with irradiated doses than the planned values, indicating decreased homogeneity of the dose distribution to the target volumes. Even greater deviations from the planned value were observed in the CI of the PTV1, which were the high-risk lymph nodular target volume, ranged from -32.45% to 13.97%, indicating that the dose conformity of PTV1 in some individual cases decreased a lot after the plan delivery, which might have led to inadequate dose coverage in this target volume or to elevated doses in the surrounding normal tissues. The CI of the PTV2 decreased by an average of approximately 2% (P < 0.05), thus showing a total reduction of the PTV2 dose conformity during the implementation of the plan.

Elmpt et al. [17] reported a work of similar 3D-ADV, using the planning CT image to reconstruct the dose distribution in combination with a Monte Carlo calculation and the energy fluence of the actual treatment beams measured pre-treatment with the electronic portal image device (EPID). In their study on head and neck cancers IMRT cases, most deviations between the reconstructed delivered dose and the planned value in the PTV, including D5%, D_mean_ and D95%, were less than 3%, while the mean dose in the parotid gland decreased by 3.2% ± 1.2% and the maximum spinal cord dose increased by 3.1% ± 1.9%. Our 3D verification data showed that the deviations in the D2%, D98% and D95% of the PTVs and the deviations in the V100% and V95% were all within ±5%, which were similar to the previous report. In contrast, the maximum deviations of D2% in the optic nerves and the optic chiasm were 31% and 27%, respectively, suggesting that extra attention should be given to future plan verifications with regard to whether the delivered dose to these organs will exceed the clinical limits. In the reported study of Stasi [18] and Carrasco [19], a weak correlations or even no correlation has been found between the gamma index and the clinical impact of a delivery dose discrepancy, like the deviation on DVH for PTV and OAR volumes. The acceptance criteria for which we had the highest frequency of correlations were (3%, 3 mm), however, this criterion hid relevant clinical dose metric differences which is not clinically acceptable. Our study also shown that there might be clinically unacceptable dose discrepancy in some cases even the (3%, 3 mm) gamma pass rate was very high.

Traditional 2D-PDV is relatively simple and easy to perform. However, this method cannot provide information regarding anatomical positions and the dose volumes that correspond to the dose deviations; thus, it can only serve as a basic quality assurance tool for IMRT. using online measured results, 3D-ADV is able to reconstruct the dose distributions from patients' anatomical images and provide us with more clinical relative information, verify the delivery deviation in both dosimetric and geometric parameters in the same way of plan evaluation. In the other hand, unlike 2D-PDV devices, the 3D-ADV utilized an independent calculation algorithm in the dose reconstruction, which differed from that used in the planning system. Therefore, it might lead to additional discrepancy in the verification results, if the dose reconstruction computation of the 3D-ADV was not accurate enough. It is important that a very strict pre-commissioning and proper evaluation has been performed when the 3D-ADV system is used for the clinic treatment plan QA.

## Conclusion

In this study of 20 nasopharyngeal carcinoma patients who were treated with IMRT or VMAT, comparing the results of the delivered dose distributions from traditional 2D-PDV and the 3D-ADV, it was confirmed that a relatively large local dose deviation might exist in the delivered dose distributions, even when the global gamma pass rate is very high in both verification. A 3D-ADV, providing structure by structure volumetric dose evaluation is suggested to be used as further clinical quality assurances for IMRT/VMAT therapy of complex cases like nasopharyngeal carcinoma.

## Competing interests

The authors declare that they have no competing interests.

## Authors’ contributions

HL performed this study as part of his Master thesis. SH assisted with the treatment planning and dosimetry measurements. XD conceived of the study, participated in its design and coordination and helped to draft the manuscript. JZ assisted with the computation program of angular correction for ion-chamber array. LC helped to perform the measurements and data analysis. All authors reviewed and approved the final manuscript.
